# Feasibility of Deploying Home-Based Digital Technology, Environmental Sensors, and Web-Based Surveys for Assessing Behavioral Symptoms and Identifying Their Precipitants in Older Adults: Longitudinal, Observational Study

**DOI:** 10.2196/53192

**Published:** 2024-05-08

**Authors:** Wan-Tai M Au-Yeung, Yan Liu, Remonda Hanna, Sarah Gothard, Nathaniel Rodrigues, Cierra Leon Guerrero, Zachary Beattie, Jeffrey Kaye

**Affiliations:** 1 Oregon Health & Science University Portland, OR United States; 2 Fariborz Maseeh Department of Mathematics + Statistics Portland State University Portland, OR United States

**Keywords:** neuropsychiatric symptoms, mild cognitive impairment, dementia, unobtrusive monitoring, digital biomarkers, environmental precipitants, mobile phone

## Abstract

**Background:**

Apathy, depression, and anxiety are prevalent neuropsychiatric symptoms experienced by older adults. Early detection, prevention, and intervention may improve outcomes.

**Objective:**

We aim to demonstrate the feasibility of deploying web-based weekly questionnaires inquiring about the behavioral symptoms of older adults with normal cognition, mild cognitive impairment, or early-stage dementia and to demonstrate the feasibility of deploying an in-home technology platform for measuring participant behaviors and their environment.

**Methods:**

The target population of this study is older adults with normal cognition, mild cognitive impairment, or early-stage dementia. This is an observational, longitudinal study with a study period of up to 9 months. The severity of participant behavioral symptoms (apathy, depression, and anxiety) was self-reported weekly through web-based surveys. Participants’ digital biomarkers were continuously collected at their personal residences and through wearables throughout the duration of the study. The indoor physical environment at each residence, such as light level, noise level, temperature, humidity, or air quality, was also measured using indoor environmental sensors. Feasibility was examined, and preliminary correlation analysis between the level of symptoms and the digital biomarkers and between the level of symptoms and the indoor environment was performed.

**Results:**

At 13 months after recruitment began, a total of 9 participants had enrolled into this study. The participants showed high adherence rates in completing the weekly questionnaires (response rate: 275/278, 98.9%), and data collection using the digital technology appeared feasible and acceptable to the participants with few exceptions. Participants’ severity of behavioral symptoms fluctuated from week to week. Preliminary results show that the duration of sleep onset and noise level are positively correlated with the anxiety level in a subset of our participants.

**Conclusions:**

This study is a step toward more frequent assessment of older adults’ behavioral symptoms and holistic in situ monitoring of older adults’ behaviors and their living environment. The goal of this study is to facilitate the development of objective digital biomarkers of neuropsychiatric symptoms and to identify in-home environmental factors that contribute to these symptoms.

## Introduction

Neuropsychiatric symptoms (NPSs) are experienced by most people living with mild cognitive impairment (MCI) and people living with dementia [1,2]. NPSs include apathy, anxiety, depression, agitation, wandering, psychosis, and sleep disturbance [1]. It is estimated that NPSs affect up to 90% of all people living with dementia over the course of their illness. Even in the early stages of cognitive impairment, NPSs are frequent, with estimated rates of 35% to 85% in people living with MCI [1] and are independently associated with poor outcomes including distress among patients and caregivers, long-term hospitalization, misuse of medication, and increased health care costs [3-5]. For people living with MCI, apathy, depression, and anxiety are the most commonly observed NPSs, each of which have been linked to cognitive and functional decline in daily activities and disease progression [6].

Currently, a major challenge in the assessment of NPSs is that they are assessed subjectively, briefly, and episodically by people living with MCI, people living with dementia, or caring partners [7-12]. As subjective data can be prone to bias [13] and these symptoms may wax and wane in severity, current methods of self-reports or infrequent queries may misidentify or miss the emergence or changes in these symptoms over time. Using digital biomarkers (defined as “objective, quantifiable, physiological, and behavioral data that are collected and measured by means of digital devices, such as embedded environmental sensors, portables, wearables, implantables, or digestibles” [14]), one may begin to address these limitations by providing more continuous and objective measurements of behaviors that are signatures of these NPSs in real-life settings. Within this context, the overall goal of this study is to develop objective, largely passive, and ecologically valid digital biomarkers of the NPSs among older adults, focusing in particular on prevalent apathy, depression, and anxiety. The findings reported in this paper address the feasibility of capturing digital biomarkers and examining how they relate to the reported measures of apathy, depression, and anxiety. The development of these behavioral, digital biomarkers may reduce subjectivity in the assessment of these symptoms; enable continuous monitoring of these symptoms; and reduce the survey burden of people living with MCI, people living with dementia, or their caregivers. As NPSs such as apathy, depression, and anxiety often precede the diagnosis of MCI or dementia [15-17], effective and continuous monitoring of such symptoms can enable the early detection of MCI or dementia, so that timely treatments may be initiated or maintained. Behavioral digital biomarkers may also reduce the sample size needed in clinical trials as shown in previous studies [18,19].

In addition to more objectively and continuously assessing NPSs, digital technologies may also have an important role in identifying the precipitants of NPSs. In particular, environmental conditions (eg, light, noise, and temperature) may be stimulating or mediating factors in the emergence of NPSs [20-22]. Therefore, including environmental sensing technology in the home technology assessment suite may provide additional, important, linked information in the eventual assessment and treatment of NPSs, because the environment may be readily modified if any of these factors are found to play a role in stimulating or sustaining NPSs. Importantly, intelligent environmental manipulation or modification that mediates NPSs can be achieved at low cost and could be implemented widely. This can potentially decrease the amount of pharmacological intervention administered to patients, which are frequently associated with adverse side effects. Thus, one of the major aims of this study is to develop and integrate environmental sensing into the digital biomarker assessment suite and identify the possible associations of NPSs with environmental conditions at home or in the community.

Currently, most of the literature is focused on developing digital biomarkers and finding environmental precipitants of NPSs in people living with moderate- to later-stage dementia, whereas those for people living with MCI or early-stage dementia have been insufficiently examined. At the same time, to date, most studies that measured the physical environment were conducted in long-term, assisted living units. The few studies that were conducted at homes neither deployed objective, physical environmental sensors [23] nor focused on apathy, depression, or anxiety [24]. This study addresses a critical need as addressing these NPSs early on may slow disease progression and help people live independently for a longer period in their home. This study examines the feasibility of deploying a weekly web-based survey in addition to deploying an in-home behavioral and environmental assessment suite in the homes of older adults who may have these NPSs.

## Methods

### Ethical Considerations

Ethics approval was obtained from Oregon Health and Science University (OHSU) institutional review board (IRB; approvals 24210, 2765, and 20236).

To protect participants’ privacy during recruitment, consent, and study procedures, participants were assigned a code that were used instead of their name or any personally identifying information. Electronic files for data analysis only contained the participant code. The key associating the codes and the participants personally identifying information was restricted to the PI and study staff. The key was kept secure on a restricted OHSU network drive in a limited access folder. Any documents that contained personally identifying information were handled according to standard institutional practices at OHSU to maintain the confidentiality and security of data. The study database was password-protected. Data were encrypted and personal identifying information was excluded from data uploads to prevent interception of data during broadband transmission. Firewall protection and password-restricted access protected against unauthorized access to the central server.

### Study Design and Overview

This is an observational, longitudinal study (OHSU IRB 24210) with a duration of 9 months. Study participants are men or women recruited from the Digital Technology Core (DTC) of the Oregon Alzheimer’s Disease Research Center (OADRC; OHSU IRB 2765) and the Oregon Roybal Center for Care Support Translational Research Advantaged by Integrating Technology (ORCASTRAIT; OHSU IRB 20236). When participants are first enrolled into the DTC or ORCASTRAIT, they are assessed in person using standardized health, behavioral, and cognitive tests (Table 1).

**Table 1:** List of standardized health, behavioral, and cognitive tests that are used to assess participants in the Digital Technology Core (DTC) or Oregon Roybal Center for Care Support Translational Research Advantaged by Integrating Technology (ORCASTRAIT) when they enroll.

Tests			DTC		ORCASTRAIT
**UDS^a^—required forms**					
	A1—Subject Demographics	✓		✓	
	A2—Informant Demographics	✓		✓	
	A3—Subject Family History	✓		✓	
	A4—Subject Medications	✓		✓	
	A5—Subject Health History	✓		✓	
	B4—Global Staging CDR^b^	✓		✓	
	B5—Neuropsychiatric Inventory Questionnaire	✓		✓	
	B6—Geriatric Depression Scale	✓		✓	
	B7—Functional Assessment Questionnaire	✓		✓	
	B8—Neurological Examination Findings	✓		✓	
	B9—Clinician Judgement of Symptoms	✓		✓	
	C2—Neuropsychological Battery Scores	✓		✓	
	MoCA^c^ or Blind MoCA version	✓		✓	
	Craft Story 21 (immediate)	✓		✓	
	Benson Complex Figure Copy (immediate)	✓		✓	
	Number Span Forward	✓		✓	
	Number Span Backward	✓		✓	
	Category Fluency (animals)	✓		✓	
	Category Fluency (vegetables)	✓		✓	
	Trail Making Test (parts A and B) or Oral Trail (parts A and B)	✓		✓	
	Craft Story 21 (delayed)	✓		✓	
	Benson Complex Figure Recall	✓		✓	
	Multilingual Naming Test	✓		✓	
	Letter Fluency (F, L, and C)	✓		✓	
	D1—Clinician Diagnosis	✓		✓	
	D2—Clinician-assessed Medical Conditions	✓		✓	
CERAD^d^ Word List			✓		✓
Visual Reproduction 1 and 2 (WMS-R^e^)			✓		
Digit Symbol			✓		
Stroop Test			✓		
Block Design (WAIS-R^f^)			✓		
Oregon Gait and Balance Inventory			✓		
OARS ADL or IADL^g^			✓		✓
Collateral Clinical Dementia Rating Interview Questions			✓		
Collateral Clinical Dementia Rating Scale			✓		
QOL-AD^h^					✓
Lubben Social Network Scale					Web based
GAD-7^i^					✓
Adverse Childhood Experience					✓
Caregiving Annual Questions (CP^j^ only)					✓
Dyadic Relationship Scale (CP only)					Web based
Zarit Burden Interview (CP only)					Web based
Weekly questionnaire			Web based		Web based

^a^UDS: Uniform Data Set.

^b^CDR: Clinical Dementia Rating.

^c^MoCA: Montreal Cognitive Assessment.

^d^CERAD: Consortium to Establish a Registry for Alzheimer’s Disease.

^e^WMS-R: Wechsler Memory Scale-Revised.

^f^WAIS-R: Wechsler Adult Intelligence Scale-Revised.

^g^OARS ADL or IADL: Older Americans Resources and Services Activities of Daily Living or Instrumental Activities of Daily Living.

^h^QOL-AD: Quality of Life-Alzheimer’s Disease.

^i^GAD-7: General Anxiety Disorder-7.

^j^CP: care partner.

All participants are sent a weekly web-based survey querying about their health and activity measures [25] and have a technology platform deployed in their homes that was developed by the Oregon Center for Aging and Technology (ORCATECH) [26,27]. When participants consent to participate in the study described in this paper (OHSU IRB 24210), they also agree to complete a weekly web-based survey for assessing their level of apathy, depression, and anxiety in the past week. The weekly queries are web based, allowing for responding through any internet-connected device (eg, smartphone, tablet, laptop, or PC). Participants are not provided with a device but use their own internet-connected devices to complete the surveys. In addition, participants of this study had an environmental sensor added to the ORCATECH home assessment platform to measure the indoor environment of their residence.

### Participants

Study coordinators reach out to potential participants in the OADRC DTC and ORCASTRAIT study cohorts through phone calls or emails to gauge their interest and eventually obtain informed consent for participation in this study. Participants have the option of consenting electronically (e-consent) or using paper consent forms. An individual is considered capable of consent if they have a score ≤0.5 on the Clinical Dementia Rating scale [28]. If not capable, the assent of the participant is required and is obtained by explaining the study to the participant and having the participant sign the consent form along with their legally authorized representative. The target population is older adults aged ≥60 years without major comorbidity. People with stable, common, age-associated medical conditions (eg, hypertension, osteoporosis, and osteoarthritis) are not excluded. Participants may have normal cognition, MCI, or early-stage dementia diagnosed using National Alzheimer’s Coordinating Center criteria [29]. They may or may not have symptoms of apathy, depression, or anxiety as assessed using the Uniform Data Set Form B5: Neuropsychiatric Inventory Questionnaire or Uniform Data Set Form B9: Clinician Judgment of Symptoms [29]. The goal is to recruit 6 participants with normal cognition and 6 participants with either MCI or early-stage dementia. As apathy, depression, and anxiety are highly prevalent in people living with MCI or early-stage dementia and less common in people with normal cognition, the expectation is to recruit a sample of participants with and those without the NPSs of interest.

### Weekly NPS Assessment

Participants are sent an email every Monday morning at 9:00 AM, which contains a link to a web-based survey (Qualtrics) that assesses their level of behavioral symptoms including apathy, depression, and anxiety in the past 7 days. Each symptom is assessed using a distinct assessment survey that is placed on a separate web page, and the participants can leave any questions unanswered. Apathy is assessed using the Withdrawal-Apathy-Vigor (WAV) subscale from the 30-item Geriatric Depression Scale [30,31]. WAV scores range from 0 to 6, with 0 indicating no apathy and 6 indicating the most severe apathy. WAV has been shown to be congruent with disengagement or depletion [30]. Depression and anxiety were assessed using the 4-item depression and anxiety subscales from the 29-item Patient-Reported Outcomes Measurement Information System [32]. The possible scores for the weekly assessment of each symptom subscale range from 4 to 20, with a score of 4 being the mildest and a score of 20 being the most severe. These two 4-item subscales have been shown to have good internal reliability and convergent validity [33]. In addition, all 3 subscales have the advantage of being brief, so they should not be very burdensome to participants. In addition to the weekly web-based assessment of the 3 NPSs, a 13-item questionnaire is also sent weekly to assess other relevant health-related and activity-related events and experiences including pain, loneliness, illness, falls, visits to the emergency department or hospital, medication changes, and the need for additional assistance at home [25]. The participants receive no training for completing the surveys.

### Digital Technology

As part of the OADRC DTC or ORCASTRAIT cohort participation, all participants have the ORCATECH home assessment platform deployed in their residences. The platform includes the following: activity sensing watches (Steel; Nokia or Activité; Withings), sleep and nighttime activity sensing bed mats (ENM-9360-0656-30-P; Emfit), electronic pillboxes (TimerCap), passive infrared (PIR) motion sensors (NYCE), and contact sensors (NYCE). Participants are instructed to wear the activity sensing watch on their wrist to measure their number of steps. The bed mat is placed underneath the mattress of each participant and provides sleep measurements such as total sleep duration, sleep latency, or total duration that the participant is awake after having initially fallen asleep. The bed mat also measures physiological metrics such as heart rate and respiratory rate. The motion sensors are installed in each living space (eg, kitchen, living room, bedrooms, and bathrooms) to detect the participant’s presence in each living space. Contact sensors are placed on egress doors of a participant’s home to detect door openings and closings. More details regarding the ORCATECH technology platform can be found in the paper by Beattie et al [27]. All the digital technologies were designed to be as unobtrusive as possible and require minimal input from a participant. The ORCATECH home assessment platform is deployed in participant homes for the duration of the study.

A previous study showed that apathy was associated with physical inactivity in community-dwelling older adults [34]. Moreover, the literature suggests that sleep disturbances are associated with anxiety and depression in older adults [35]. With the digital technology in the ORCATECH platform, activity level and sleep can be measured objectively, and they can be compared against the self-reported severity of behavioral symptoms to identify correlation.

### Environmental Sensing

After consenting to participate in this study (OHSU IRB 24210), an environmental condition sensing device (Awair Omni; Awair) is added to the ORCATECH home assessment platform during a scheduled visit from a study technician to the participant’s home. The environmental sensor is placed at shoulder height on the wall of the room where the participant typically sleeps. The Awair Omni is deployed for the entire duration of the study and is connected to the internet via the ORCATECH platform hub computer in the home. The Awair Omni measures ambient light level (lux), noise level (dB), temperature (°C), relative humidity (%), carbon dioxide level (parts per million), total volatile organic compounds (parts per billion), and particulate matter–2.5 (PM2.5; µg/m^3^) every 5 minutes. The Awair Omni is an indoor RESET Air [36] Accredited-Grade B monitor for carbon dioxide, total volatile organic compounds, relative humidity, temperature, and PM2.5. The Awair Omni also generates a metric (*Score*), which quantifies the air quality and ranges from 0 to 100, with a higher number indicating better air quality. Participants’ indoor environmental data are downloaded from the Awair company website. The Awair Omni device was chosen because it measures multiple physical environmental variables that may be associated with apathy, depression, and anxiety in older adults. Light may reduce depressive symptoms in older adults and improve their quality of life [37]. Noise is associated with more severe anxiety [38]. Air quality has also been suggested to be associated with depressive and anxiety symptoms in older adults [39]. The Awair Omni device also collects data passively and requires no interaction from the participant.

### Data Analysis

We addressed the feasibility of deploying the web-based weekly surveys, the digital technology, and the environmental sensing tool by examining protocol adherence and the amount of missing data. Answers to weekly surveys were summarized through descriptive statistics. In addition, we explored how digital biomarkers from the technology platform related to the reported measures of NPSs and identified the possible associations between NPSs and environmental conditions at homes. Weekly means were calculated for the digital biomarkers and environmental data. These statistics were compared to the self-reported level of NPSs using Spearman correlation coefficients intraindividually.

## Results

### Clinical Characteristics of Participants

At 13 months after recruitment began, a total of 9 participants had enrolled into the study (Table 2). At that time, participant 1 had withdrawn after 7.5 months, participants 3 and 5 have completed the 9-month study, and the remaining 67% (6/9) of the participants were actively participating in the study. The average age of participants at enrollment was 76.3 (SD 5.10) years, 56% (5/9) are men, and 44% (4/9) are women. Participants had 17.6 (SD 3.5) years of education and, to date, have 219 (SD 46.3) days of follow-up. In terms of cognition, of the 9 participants, 5 (56%) have normal cognition, 3 (33%) have MCI, and 1 (11%) has early-stage Alzheimer disease. The Montreal Cognitive Assessment–BLIND score for the 9 participants at baseline was 19 (SD 1.80). Of the 9 participants, 1 (11%) had all 3 NPSs at baseline, 3 (33%) had none, and the other 5 (56%) had either 1 or 2 of the NPSs being studied.

**Table 2:** Summary of participants’ demographics and the presence of behavioral symptoms during their baseline clinical assessments.

Participant	Cognition	Sex	Age (y)	Race	Years of education	Behavioral symptoms reported during baseline assessment (UDS^a^–B5: Neuropsychiatry Inventory Questionnaire or UDS–B9: Clinician Judgment of Symptoms)		
						Apathy	Depression	Anxiety
1	Early Alzheimer disease	Male	80	White	18	Y^b^	Y	Y
2	Normal cognition	Female	80	White	16	N^c^	N	N
3	MCI^d^	Male	80	White	18	N	N	Y
4	Normal cognition	Female	71	White	16	N	N	N
5	MCI	Male	69	White	25	Y	N	N
6	MCI	Male	74	White	19	Y	Y	N
7	Normal cognition	Male	71	White	16	N	Y	N
8	Normal cognition	Female	83	Black	12	N	N	N
9	Normal cognition	Female	79	White	18	N	N	Y

^a^UDS: Uniform Data Set.

^b^Y: symptoms reported at baseline.

^c^N: no such symptom reported at baseline.

^d^MCI: mild cognitive impairment.

### Web-Based Weekly Surveys

Table 3 summarizes the weekly surveys, both at the individual level and group level. Among a total of 278 web-based questionnaires sent, only 4 (1.4%) were not even started. The remaining were either fully completed (241/278, 86.7%) or partially completed (33/278, 11.9%).

**Table 3:** Weekly survey adherence and neuropsychiatric symptom score statistics.

Participant	Weekly surveys sent, n^a^	Responses received, n^b^	Apathy score, mean (SD)^c^	Depression score, mean (SD)^d^	Anxiety score, mean (SD)^e^
1	32	32	1.13 (0.942)	5 (1.04)	5.29 (1.65)
2	23	23	0 (0)	4.24 (0.526)	4.14 (0.343)
3	40	40	2.03 (0.357)	4 (0)	4.03 (0.156)
4	32	32	2.06 (1.01)	4.25 (0.612)	4.22 (0.544)
5	39	39	5.00 (0.392)	10.6 (0.718)	10.5 (0.873)
6	35	32	5.97 (0.174)	14.3 (1.46)	13.5 (1.60)
7	24	24	0.625 (1.03)	5.92 (0.400)	4 (0)
8	30	30	1.47 (0.806)	5.45 (0.723)	5.97 (1.02)
9	23	23	0 (0)	6.74 (1.36)	5.23 (0.849)

^a^Group mean 30.9 (SD 6.53).

^b^Group mean 30.6 (SD 6.37).

^c^Group mean 2.47 (SD 2.09).

^d^Group mean 6.82 (SD 3.50).

^e^Group mean 6.56 (SD 3.45).

**Figure 1:**
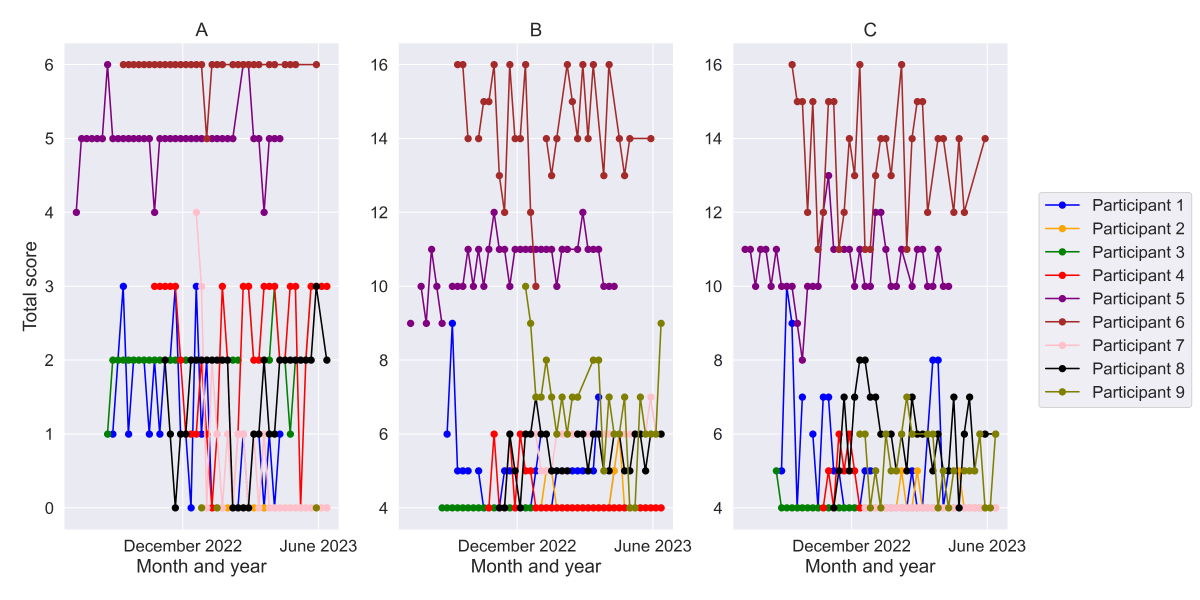
Total (A) apathy, (B) depression, and (C) anxiety scores reported on the weekly web-based questionnaire over time for 9 participants, indicating intraindividual and interindividual variability in neuropsychiatric symptom severity.

Responses to the weekly queries regarding apathy, depression, and anxiety were highly variable both across and within individuals over time (Figure 1). Of the 9 participants, 2 (22%; participant 5 and participant 6) were characterized as having higher apathy, depression, and anxiety scores over time compared to the rest of the cohort. Both these individuals had MCI at baseline. Participant 5 had symptoms of apathy at baseline, and participant 6 had symptoms of apathy and depression at baseline.

### Adherence to and Feasibility of Data Collection Using Digital Technology and Environmental Sensor

All participants had PIR motion and contact sensors successfully deployed in their homes. All participants except participant 1 (8/9, 89%) were given an electronic pillbox. Participant 1’s home had connectivity issues with the electronic pillbox device and thus did not have the device when enrolled in this study. Of the 9 participants, 7 (78%) wore the activity sensing watches. Participants 1 and 2 did not wear them. Participant 1 had their own commercial wearable and preferred that to the study’s activity sensing device. Participant 2 found wearing the activity sensing device uncomfortable. All participants except participant 6 (8/9, 89%) had bed pressure mats installed. Participant 6 reported the bed pressure mat as being very intrusive.

Table 4 shows the number and percentages of days for which data were collected from the respective device and each participant’s home over their follow-up period. At the sample level, over the mean follow-up of 219 days, the PIR motion and contact sensors captured data for an average of 196 (89.5%) days, the bed mats captured data for 157 (71.7%) days, the activity sensing watch captured data for 164 (74.9%) days, and the electronic pillbox captured data for 165 (75.3%) days. The Raspberry Pi (hub computer in each home) occasionally went offline, requiring a restart that accounted for most data outages. Participant 3 moved during the study, which accounted for some of the data outages for their home. On other occasions, the devices themselves were disconnected from the Wi-Fi or lost communication with the hub computer. Other times, data were not collected because participants did not use the devices. For example, when participants went out of town or did not sleep on their beds, then no data are collected from the bed mat. In addition, participants would occasionally forget to wear the activity sensing watches in the morning, so no data were collected throughout that day. It is not possible to identify the reason for every data loss. For devices that participants interact with, only the medication adherence tracking devices would automatically confirm that they were functioning every day regardless of whether the participants used them. If the medication adherence tracking devices confirmed that it was functioning, but no data were collected regarding participants’ uses, then it can be assumed that the participants did not use the devices. However, if there is no activity sensing watch or bed mat data, it can be difficult to know whether they are not being used or whether there was a technical issue. As the motion and contact sensors would also check in every day, it was possible to know whether they were working.

**Table 4:** Number and percentage of days with data for each digital technology.

Participant	Follow-up period (days), N	Bed mat, n (%)^a^	Motion and contact sensor, n (%)^a^	Activity sensing watch, n (%)^a^	Medication adherence tracking device, n (%)^a^	Days Awair Omni was deployed, n	Awair Omni, n (%)^b^
1	225	195 (86.7)	163 (72.4)	N/A^c^	N/A	186	182 (97.8)
2	161	146 (90.7)	161 (100)	N/A	155 (96.3)	160	159 (99.4)
3	274	193 (70.4)	228 (83.2)	232 (84.7)	80 (29.2)	197	174 (88.3)
4	224	203 (90.6)	223 (99.6)	217 (96.9)	204 (91.1)	210	208 (99)
5	275	243 (88.4)	244 (88.7)	258 (93.8)	255 (92.7)	254	248 (97.6)
6	264	N/A	206 (78)	264 (100)	262 (99.2)	243	196 (80.7)
7	162	141 (87)	162 (100)	162 (100)	161 (99.4)	159	158 (99.4)
8	210	185 (88.1)	201 (95.7)	191 (90.9)	190 (90.5)	202	201 (99.5)
9	172	106 (61.6)	172 (100)	150 (87.2)	172 (100)	128	111 (86.7)

^a^Denotes the number of days with data and the percentage of days with data over the follow-up period.

^b^Denotes the number of days with data over the number of days deployed and the percentage of days with data over the number of days deployed.

^c^N/A: not applicable.

The Awair Omni environmental sensors were deployed in all homes (9/9, 100%). The number of days the Awair Omnis were deployed were shorter than the study duration because they were added after participants had consented to participate in this study and it took time for the study staff to schedule home visits to install these sensors. An average of 90.4% (SD 10%) of environmental data were successfully collected from all homes during the monitoring periods when the Awair Omnis were marked as active. There were 2 occasions when an Awair Omni was marked as inactive. One occasion was due to construction at the participant’s home, and the other was due to the participant moving. The Awair Omni would become disconnected from the internet occasionally. This issue was often resolved by power cycling the device. However, this did not always lead to data loss because the environmental data can be stored locally on the device for up to approximately 10 days even when it is disconnected from Wi-Fi.

**Figure 2:**
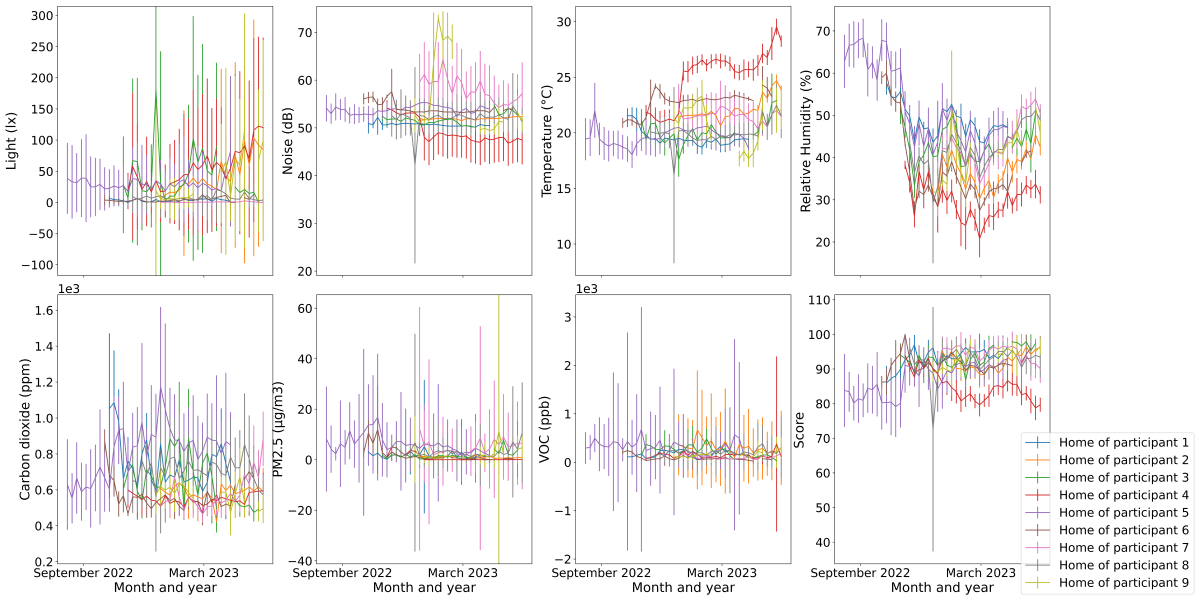
Weekly mean environmental data along with error bars showing the SDs at participants’ homes throughout their follow-up periods. ppm: parts per million; ppb: parts per billion; PM2.5: particulate matter–2.5; VOC: volatile organic compound.

Figure 2 shows the environmental data from each participant’s home. There was time-dependent heterogeneity across homes in all the environmental domains measured. The PM2.5 levels were highest in the homes of participants 5 (purple) and 6 (brown) in October 2022, which were time aligned with the wildfires occurring during that time in Oregon. Moreover, there was seasonality in the relative humidity data. As participants 5 and 6 had the longest follow-up periods for their in-home environment, the humidity level was lower during winter compared to summer and autumn, which is contrary to the outdoor environment. This was likely due to participants switching on their heaters during winter. In addition, by examining the data shown in Figure 2, it was noticeable that the environment data for participant 4’s home changed considerably, starting from the beginning of 2023. It is speculated that the environmental sensor at this home had not been working properly since 2023 as the daily PM2.5 level dropped to approximately 0 µg/m^3^, which is extremely low.

### Exploratory Data Visualization and Analysis

**Figure 3:**
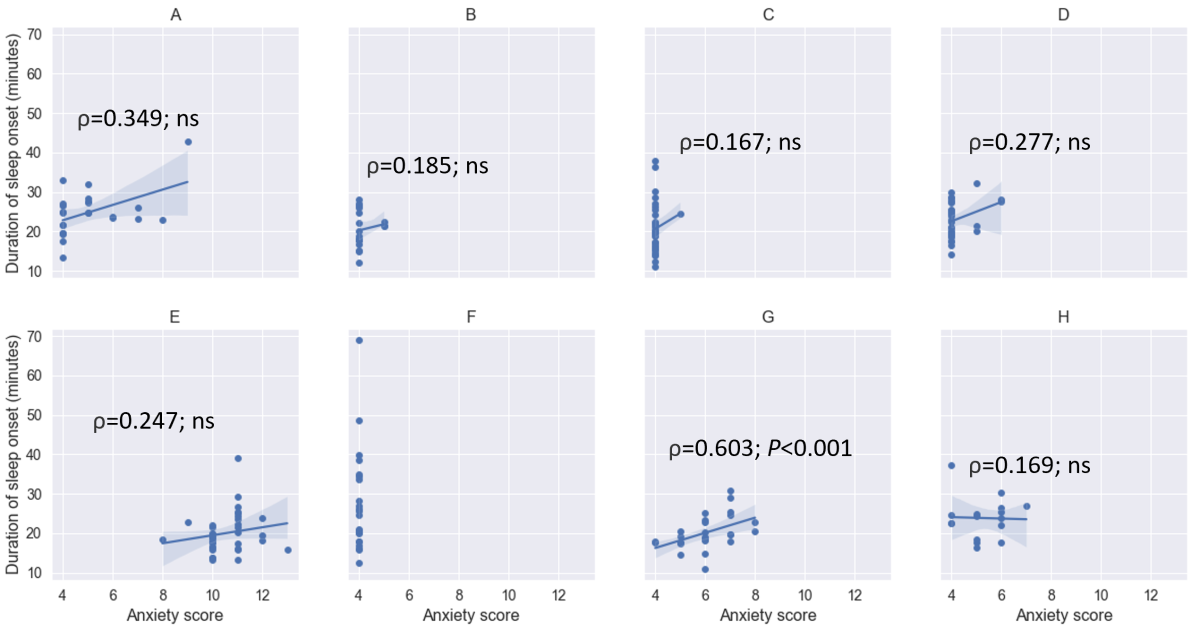
Scatter plots of duration of sleep onset in minutes versus self-reported level of anxiety in weekly surveys for (A) Participant 1, (B) Participant 2, (C) Participant 3, (D) Participant 4, (E) Participant 5, (F) Participant 7, (G) Participant 8 and (H) Participant 9. Some individuals had little or no change in the levels of anxiety (participants 2, 3, and 7), whereas others had a wide range of change in anxiety or duration of sleep onset. ns: not significant.

In general, there were large variations in NPSs and the related digital biomarker signals (eg, sleep measures and step counts). For example, the within-individual correlation between duration of sleep onset and self-reported level of anxiety is especially noteworthy. The mean duration of sleep onset for the 89% (8/9) of the participants who had bed pressure mats was 22.3 (SD 6.84) minutes. The mean duration of sleep onset for each individual participant ranged from 20 to 27.7 minutes. Figure 3 shows the scatter plots of duration of sleep onset from the bed mats versus self-reported level of anxiety grouped based on participant, along with the corresponding Spearman correlation coefficients (ρ) and statistical significance. As shown by the correlation coefficients, durations of sleep onset were positively correlated with the self-reported levels of anxiety for all participants, with one of them being statistically significant (*P*<.001).

**Figure 4:**
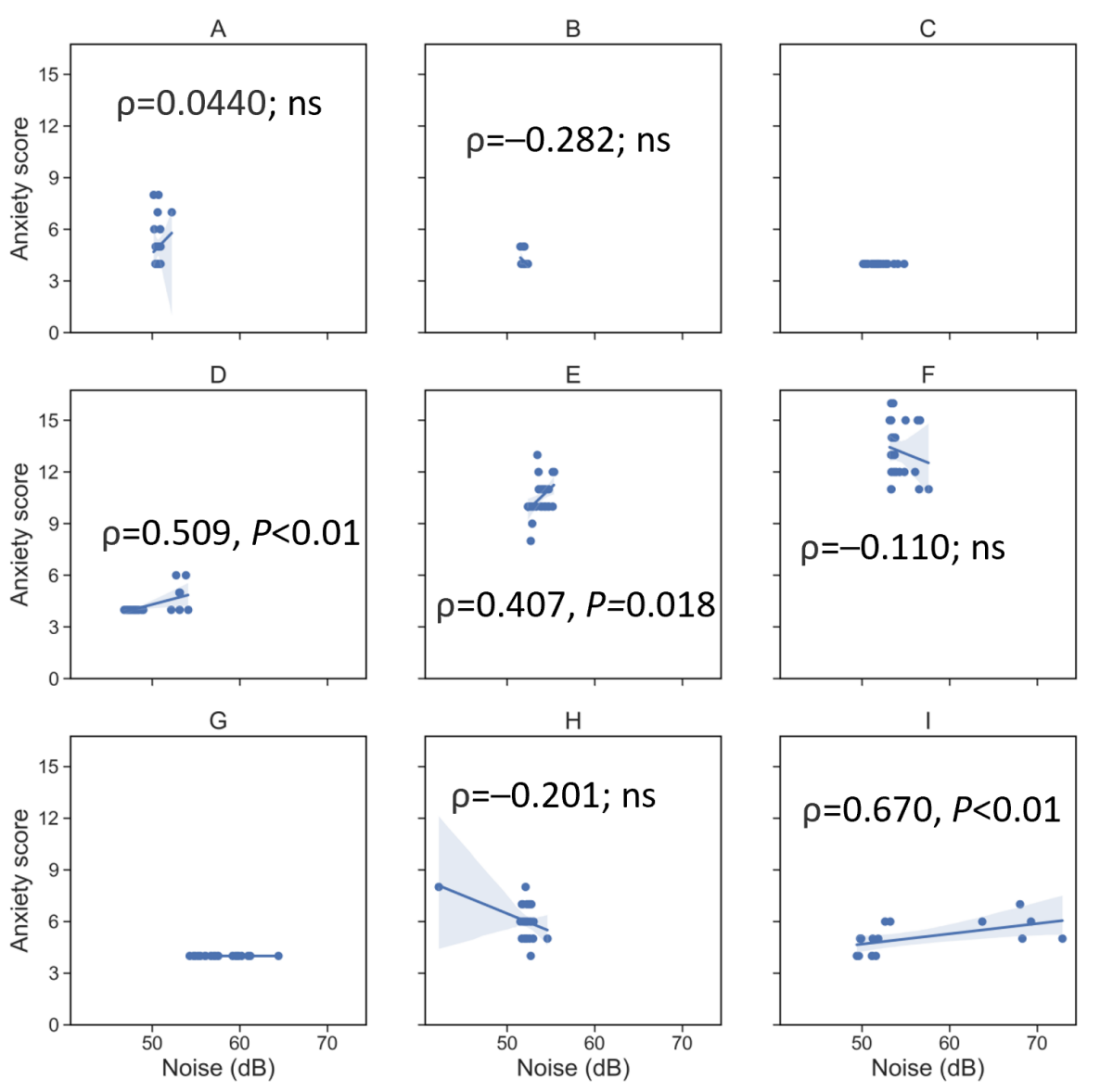
Scatter plots of self-reported level of anxiety from weekly web-based surveys versus weekly mean noise level (dB) at home for (A) Participant 1, (B) Participant 2, (C) Participant 3, (D) Participant 4, (E) Participant 5, (F) Participant 6, (G) Participant 7, (H) Participant 8 and (I) Participant 9. The corresponding Spearman correlation coefficients (ρ) and *P* values are provided. ns: not significant.

Figure 4 presents an example of how an environmental condition (noise level per participant home) may relate to an NPS (anxiety). Average weekly noise levels were typically >50 dB, which is above the level of a typical quiet room [40]. Anxiety was significantly and positively correlated with the noise level for 33% (3/9) of the participants (*P*=.02 for participant 5, and *P*=.005 for participants 4 and 9). Due to the abovementioned suspected technical difficulties with the Awair Omni in the home of participant 4, the observed noise levels <50 dB and the observed correlation with anxiety should be considered with a degree of uncertainty. For the other 44% (4/9) of the participants who had variation in their weekly levels of anxiety, the weekly indoor noise levels were negatively but nonsignificantly correlated with their weekly levels of anxiety.

## Discussion

### Principal Findings

In general, the sensing platform including the environmental sensing device were successfully deployed to homes despite occasional nonadherence and data loss. NPS levels of participants show week-to-week fluctuations even though the within-person variability of level of symptoms is small compared to the possible range of the level of symptoms. The study and its recruitment are ongoing, with the current duration of follow-up relatively limited. The planned collection of up to 40 weeks of data from each participant will assist in providing a wider spectrum of possible NPS changes over time and will guide the study durations for future studies. Nevertheless, the weekly collection of data about the participants’ levels of NPSs, digital biomarkers, and indoor home environments allows us to examine their changes over time and correlations among them both intraindividually and interindividually.

Participants’ levels of NPSs may be correlated with their adherence rate of completing the weekly survey as evidenced by participant 6 who had the highest level of all 3 symptoms and the lowest response rate among the 9 participants. When a person has these NPSs, they may consider completing surveys a nuisance and burdensome. This emphasizes the importance of being able to measure their level of NPSs objectively and unobtrusively.

The in-home environmental data collected in this study emphasize the importance of collecting such data because the in-home environment often does not align with the outdoor environment [41]. The temperature and relative humidity data are such examples. With older adults spending most of their time at home, indoor environment monitoring will provide a more accurate estimate about their environmental exposure. However, indoor environment sensing is a relatively new feature added to the ORCATECH platform, and one of the lessons learned while conducting this study was that even high-quality environmental sensor may not provide reliable and valid measurements after some time, as evidenced in the data of participant 4’s home. Parts may fail, and there is variability in the quality of sensors and their life span even when they were manufactured by the same company. Frequent monitoring of the environmental data by the study personnel will help in detecting unreliable data, so that a malfunctioning sensor may be replaced. At the same time, this may not always be achievable if the changes caused by the deterioration of sensors are small.

Adherence to using the technology is an issue for the activity sensing watch, for which 2 (22%) of 9 participants did not opt for the device provided by the study. This points to the importance of having technology that is unobtrusive as some participants may find using a wearable uncomfortable. At the same time, some participants may already have their own wearable that they prefer. One future direction in research is to let participants “bring your own device.” However, bringing their own device has its own challenges as each device will have unique specification, which will make standardizing and synthesizing the data difficult [42].

The ORCATECH platform was designed to be minimally intrusive to collect ecologically valid data from older adults as they live their daily lives. The platform does not record video nor audio, which preserves the privacy of our participants. However, some participants may still find some devices, such as bed mat, to be intrusive. As bed mat can collect valuable information about participants’ sleep and physiology, including an extensive educational session regarding study technology may increase participant comfort level with study devices, leading to higher acceptance and compliance.

Missing data are inevitable when data are collected in free-living environments as several problems can arise, ranging from power issues and internet issues to participants not using the devices. When such data are being analyzed, analytical methods that can account for missing data, such as mixed-effects models, will be especially applicable.

Cognitive impairment is strongly correlated with treatment incompliance and nonadherence as shown in a previous study on older adult patients with hypertension [43]. Cognitive impairment may contribute to device incompliance as memory problems may cause participants to forget to wear the activity sensing watches. These are issues that merit further investigation in future studies with a larger sample.

Several digital biomarkers and highly intensive environmental data were collected in this study with which multiple tests can be performed to examine the associations between digital biomarkers and level of NPSs and associations between the environment and the level of NPSs. This pilot study data can be used to generate hypotheses and to calculate the sample size for more definitive studies in the future. In this study, the preliminary data for duration of sleep onset collected using bed pressure mats are positively correlated with self-reported level of anxiety in all participants who had fluctuations in their level of anxiety (7/7, 100%), even though only 1 of them was statistically significant at the *P*<.05 level. Furthermore, the levels of anxiety for 22% (2/9) of the participants were statistically significantly and positively correlated with the noise level at their home (excluding participant 4). These preliminary and individual results suggest feasibility and agree with existing literature [38,44].

### Limitations

This study had limitations. One limitation of this observational study is that we did not record residential environmental factors that may affect the measured environmental variables experienced by our participants. Our participants likely used heaters, air conditioners, or other devices to keep the indoor environmental conditions within small ranges. Small variability in environmental conditions make it difficult to find associations between the level of NPSs and the environment. Moreover, as indoor environmental sensors are still costly, deploying them in the bedrooms only will not provide a good estimate of people’s environmental exposures when they are outside or in other parts of the home. Another limitation of this study is that despite the use of subscales from well-validated scales, the validity of them being used weekly for a maximum period of 9 months has not been proven. It is unknown whether participants would take the surveys less seriously as time passes. While recruitment is still ongoing, the sample size presented in this paper is small. Therefore, one future direction is to conduct a similar study but with a larger sample size, so that estimates of effect size can be derived. This study provides an examination of feasibility, an exploration of possible pitfalls, and pilot data that can help guide future power and sample size calculations.

### Conclusions

To conclude, this study demonstrates the feasibility of assessing the severity of behavioral symptoms weekly through web-based surveys and deploying in-home, digital assessment technologies among older adult individuals. It provides a guide for holistic, in situ, longitudinal monitoring of levels of NPSs and about how these behaviors may respond to changes in the living environment. The implications of deploying this approach include enabling early detection of these NPSs, characterizing the responses to treatment or intervention more effectively with objective measures using digital technology, reducing the sample sizes needed for clinical trials, and identifying the potential in-home environment interventions that can reduce the frequency and severity of NPSs in older adults.

## Abbreviations

DTC: Digital Technology Core
IRB: institutional review board
MCI: mild cognitive impairment
NPS: neuropsychiatric symptom
OADRC: Oregon Alzheimer’s Disease Research Center
OHSU: Oregon Health and Science University
ORCASTRAIT: Oregon Roybal Center for Care Support Translational Research Advantaged by Integrating Technology
ORCATECH: Oregon Center for Aging and Technology
PIR: passive infrared
PM2.5: particulate matter–2.5
WAV: Withdrawal-Apathy-Vigor
